# A Nonconventional Approach to Formocresol Pulpotomy

**DOI:** 10.5005/jp-journals-10005-1563

**Published:** 2018

**Authors:** Amitabha Chakraborty, Bibhas Dey, Sinjana Jana

**Affiliations:** 1-3 Department of Pediatric Dentistry, Haldia Institute of Dental Sciences and Research, Kolkata, West Bengal, India

**Keywords:** Access cavity, Formocresol, Pulpotomy

## Abstract

A different concept of formocresol pulpotomy procedure has been proposed where the formocresol pulpotomy is done with smaller access to the pulp chamber. In our study formocresol pulpotomy was carried out in 128 primary molar teeth with a limited amount of access opening, saving a bit of more tooth structure, which excludes the requirement of placement of a stainless steel crown (SSC). In 2 years follow-up period, no crown fracture was reported. One hundred fifteen teeth remain vital at the end of 2 years period of observation with the periodic clinical and radiographic review. The result shows 89.4% clinical and radiographic success of this nonconventional pulpotomy procedure that corroborates with several studies of the conventional formocresol pulpotomy procedure.

**How to cite this article:**

Chakraborty A, Dey B, Jana S. A Nonconventional Approach to Formocresol Pulpotomy. Int J Clin Pediatr Dent, 2018;11(6):490-495

## INTRODUCTION

Formocresol pulpotomy is one of the most common procedure in cases of mechanical and carious exposure in primary teeth. In a standard protocol, total pulpal roof are to be removed to carry out the procedure. The pulpal roof removal weakens the tooth structure and thereby requires a preformed crown to stabilize the tooth structure from possible crown fracture during mastication. A different approach had been proposed in this study where a successful formoresol pulpotomy procedure was done with minimum possible tooth structure removal and thereby no preformed crown will be required to protect the pulpotomized tooth. In this study, it has been observed that it is not only saving the crown structure, but also the pulpotomy procedure can be carried out taking much less time, which is an essential requirement when the child patient is uncooperative, and no sedation facility is available. The less time consuming, limited access opening procedure is also helpful when multiple pulpotomy is attempted in a single visit, especially in general anesthesia (GA), to reduce the time of keeping the patient in GA state.

## AIMS AND OBJECTIVES

The aims and objectives of the study are to evaluate the possibilities of success of a nonconventional, easier and faster procedure of formocresol pulpotomy, which also excludes the use of post pulpotomy SSC.

## MATERIALS AND METHODS

Twenty male and thirty-five female, total 55 patients were selected from the patients who are attending in pedodontic outpatient department (OPD) in Haldia Institute of Dental Sciences and Research (HIDSAR), Haldia, West Bengal, India. Prior permission to carry out the study has been taken from Principal of Haldia Institute of Dental Sciences and Research. The parents of all the children involved in this study have been explained the procedure and a written and signed informed consent has been taken from them. All children selected in this study were aged between 5 years and 7 years so that at least 2 years follow-up can be carried out. The children involved in this study were called to pedodontic OPD in HIDSAR for preoperative clinical examination. The carious teeth of those children that we plan to include in this study; were examined clinically by two doctors to ensure that pulpotomy procedure is the ideal treatment for those teeth. Teeth having a carious lesion that may be exposed while preparing cavity or the teeth having a deep carious lesion that may have early bacterial invasion to the pulp has been included in the study. Deep carious lesion with any history of severe pain or night pain has been excluded from the study. Preoperative intraoral periapical (IOPA) radiograph was taken for each tooth taken in the study to ensure that those teeth do not have any radiographical periapical or furcal involvement. All the children's radiograph was taken by a single radiographer present in our center. Two sets of radiograph were taken for each tooth changing the angle of the cone, and the radiographs were examined by two doctors before taking decision for pulpotomy. This was done to establish interexaminer and intraexaminer reliability.

The selection and treatment procedure was done over a period of 6 months. Two male and six female patients were treated under GA, as they were unable to cooperate in OPD clinical setup. Patients were recalled over the phone to attend the clinic every 3 months interval. They have also been told to report clinic immediately in case of crown fracture, the occurrence of pain and swelling and loss of restorations. Twelve patients were unable to continue total 2 years follow-up period, so total 43 patients, 20 male, and 23 female were considered in this study. The total numbers of teeth in 43 patients were 128, in which 76 teeth were 1st primary molars, and 52 teeth were 2nd primary molars. Most of the cases were having proximal caries. Only 19 teeth were involved with occlusal caries. Details of the teeth involved in this study are shown in Table 1.

**Fig 1:**
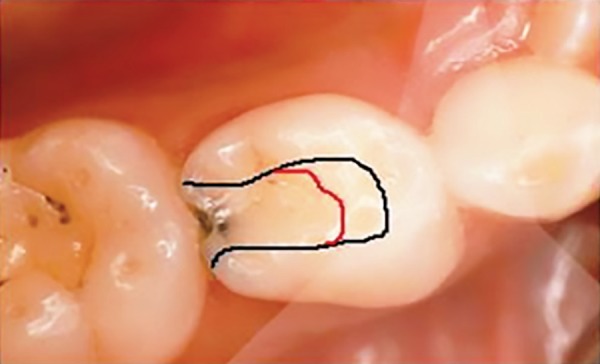
Schematic diagram of access outline in lower 1st molar. black line for conventional outline and red line for limited access outline on occlusal surface.

**Fig. 2:**
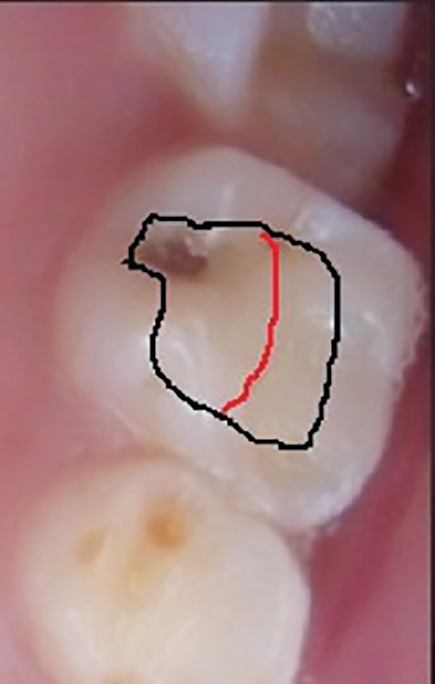
Schematic diagram of access outline in upper 2nd molar. black line for conventional outline and red line for limited access outline on occlusal surface

The conventional pulpotomy procedure involves the removal of the total roof of the pulp chamber. In this study, the objective of access opening was to preserve the tooth structure as much as possible. In case of occlusal caries at least 2/3 rd to 1/2 of the occlusal surface was attempted to be preserved. Whereas in the case of proximal caries, access is made removing approximately 1/2 of the occlusal surface (Figs 1 and 2). Any carious involving part of the crown structure was removed. Four patients were willing to accept a rubber dam application. We refrain from rubber dam application to those patients as it will create a small number of variables. Cotton role isolation was used for all of the patients who were treated in the outpatient department (OPD) clinic.

Buccal infiltration local anesthesia is given using a self-aspirating syringe with 27 gauge needle. Ten percent topical lignocaine applied over the injection area one minute before injection using the side of a cotton roll moistened with topical lignocaine spray. The pulp chamber was entered through carious lesion with a high-speed carbide round bur (H1, US No 4 by Edenta AG, Switzerland) under water cooling. Access was made up to the above-mentioned extension approximately. All cariously involved tooth, hard tissue was removed. A no 6 low-speed carbide round bur (C1S, US No 6 by Edenta AG, Switzerland) was used to rotate lightly over the inner walls of the pulp chamber in low speed with a water jet. It detached most of the pulpal attachment on the pulp chamber wall. A thorough saline wash was done into the pulp chamber to remove the coronal pulp tissue. A moist cotton pellet was plugged into the pulp chamber for a few minutes to attain hemostasis. A cotton pellet lightly moist with full strength formocresol (San Mark Dental Co. USA) was packed into the pulp chamber and kept for one minute. The radicular pulp tissue was observed for its color changes which indicate fixation. Any remaining pulp tissues over the walls of the pulp chamber were also expected to be fixed. A thick mixed zinc oxide eugenol paste was packed into the pulp chamber floor by the pressure of a cotton pellet. The access was sealed with RMGI (Vitremer, 3M ESPE) for all proximal caries and with composite resin (3M ESPE Adper easy bond and 3M ESPE Filtek composite) over an RMGI base for all occlusal caries.

**Table 1:** Position and number of teeth pulpotomized in this study following FDI two-digit notation

*Tooth No (FDI)* ➜	*54*	*64*	*74*	*84*	*55*	*65*	*75*	*85*	*Total*
*Caries* ↓									
Occlusal	02	01	03	03	02	01	04	03	19
Proximal	16	15	18	18	08	11	13	10	109
Total	18	16	21	21	10	12	17	13	128

Each patient has been told that they will be called every 3 months interval for reviewing the status of the treated tooth. Their appointment had been reminded over telephone 2–3 day before their appointment date. In every appointment, patients were reviewed clinically and an IOPA radiograph of the treated tooth as taken to see any pathological changes.

## RESULTS

**Fig. 3A to D:**
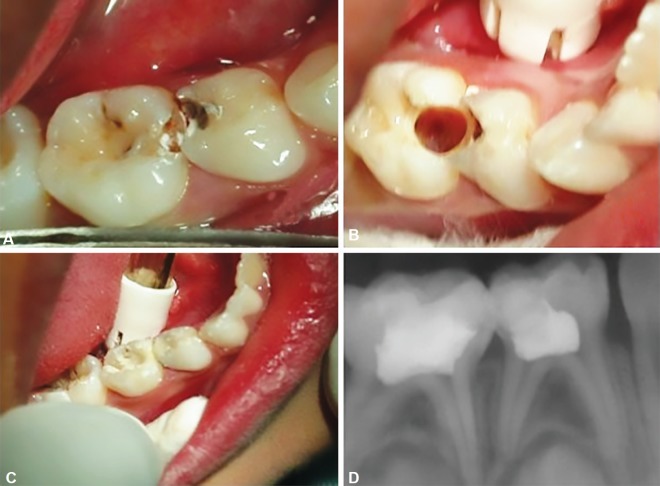
(A) Deep proximal caries in 84, 85; (B) Fixed radicular pulp of 85 using formocresol through limited access opening, Pulp chamber of 84 is not seen; (C) Post pulpotomy restoration of 84,85 using RMGI; (D) 3 months

**Figs. 4A to C:**
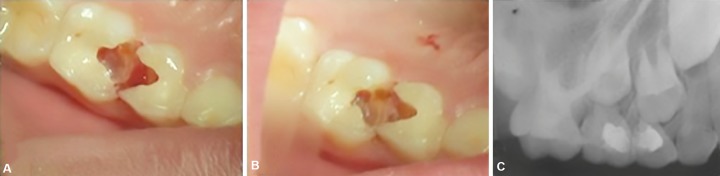
(A) Hemostasis achieved irt 64,65; (B) Fixed radicular pulp of 65. Pulp chamber of 64 is not seen; (C) 6 month postoperative radiograph

**Figs. 5A and B:**
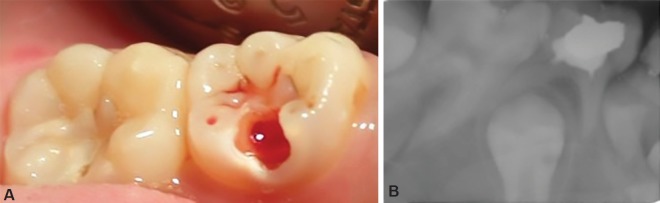
(A) Pulp chamber is opened through limited access opening in 75; (B) Immediate postoperative radiograph after pulpotomy and restoration

Each patient included in this study had been called every 3 months interval over a period of 2 years. As mentioned before, 12 patients were unable to maintain regular follow-up, and they have been excluded from the study. The patients who missed occasionally one or two appointments have not been excluded. In this two years period, no crown fracture had been observed. Thirty-two restorations were dislodged, in which 26 are RMGI restorations and 6 are composite restorations. No composite restorations have been totally dislodged, rather that were partly broken. Dislodgement of restorations is seen in patients treated in OPD clinic. Patient treated under GA did not show any dislodgement of restorations. Patients with dislodged or broken restoration reported our clinic as soon as possible, as they have been instructed in a case of lost restoration. Those teeth were refilled in the same appointment. A total of 13 teeth have shown failures to maintain vitality. One tooth that failed to retain vitality was treated under GA. Rest 12 cases of failure are the teeth that were treated under OPD clinic. Failure in second primary molar was seen in 2 cases and rest 11 failures were seen in a first primary molar. One case of failure was observed in pulpotomy of occlusal caries and rest 12 failures are cases of pulpotomy in proximal caries (Table 2). Most of the failures of keeping vitality have been seen in the first 12 months of observation. The next one year of observation has shown no more pulp vitality damage except one tooth. Two cases failed by lateral abscess formation along with bifurcation radiolucency. Four cases failed with lateral abscess formation and shown peri-apical radiolucency, and six cases failed with a history of severe pain and radiographic apical and furcalpathosis (Table 3). Rest of the teeth, i.e., 115 teeth remained vital and symptom-free during the study of 2 years. It shows almost 89.4% success rate of formocresol pulpotomy when carried out through limited access opening in this study (Figs 3 to 5).

## DISCUSSION

Formocresol was introduced by Buckley in 1904 mixing equal parts of formalin and tricresol intended to fix nonvital pulp tissue.^1^ In 1930, Sweet used so-called commercially available “Buckley's formula” with 19% formaldehyde and 35% creosol to “mummify” (fix) the pulp tissue in six visits. When fixed, the radicular pulp was expectedly “sterilized and devitalized”, thus preventing any infection and internal resorption.^2^ Later Doyle et al. started using two visit pulpotomy where formocresol was applied in the first visit. A base of zinc oxide-eugenol cement mixed with paraformaldehyde and restoration was placed in second visit.^3^ A few years later Redig reported the clinical success of a 5-minute formocresol application, and since then, the 5-minute formocresol pulpotomy protocol became the standard practice for pulpotomy.^4^ This 5-minute protocol becomes the standard against which all new modalities for pulpotomy are compared. Later Morawa^5^ after his 5 years observation, proposed a 5:1 dilution application of formocresol with a similar effect on pulp tissue like full strength formocresol. Since then it has become a gold standard of use of formocresol in formocresol pulpotomies.

Several other materials like glutaraldehyde, electro-surgery, laser, bioactive glass, etc., have been proposed for deciduous tooth pulpotomy, keeping in mind the local effect and systemic absorption of formocresol.^6–9^ All of them have shown a varied amount of success. Lately, UK National Clinical Guidelines in Pediatric Dentistry has proposed formocresol in 1:5 dilution, ferric sulfate, calcium hydroxide powder, and MTA are the choice of materials to be used for primary tooth pulpotomy.^10^ We have chosen formocresol as a material in this study because it's easy to use, less technique sensitive, huge success rate, least expansive and the choice of pulpotomy material of a greater number of pedodontist.^11–13^ Moreover till date application of formocresol is not banned by any regulatory body of any country and it is true that if judiciously used, there is no scientific and toxicological reason to abandon formocresol from dentistry.^9^ Our objective of this study is to preserve the tooth structure not the choice of material, and formocressol is the only material that can be used to fix the radicular pulp through the limited opening to the pulp chamber as the other materials recommended by UK National Clinical Guidelines does not fix the pulp tissue. As we have undertaken smaller access to the pulp chamber, it remains a chance to leave some amount of pulpal remnants on the pulp chamber wall. A formocresol application will also fix those remnants of the pulp chamber wall, and those fixed pulpal remnants will be separated from the fixed radicular pulp by compact packing of zinc oxide eugenol and glass ionomer cement in the pulp chamber. As many of the children in our study were not very much cooperative, we have undertaken a one-minute application method which also has good success rate almost the same to the 5-minute application.^14,15^ About 89.4% success rate in our study corroborates with those studies which claim the success of the one-minute application of full strength formocresol. This success rate also closely corroborates with the success rate of several studies that carried out conventional formocresol pulpotomy procedure.^16–19^

**Table 2:** Month wise treatment done and failures. Tooth indicated by FDI two digit notations

*Month of procedure*	*Number of pulpotomies done/month*	*Failure after 6 months*	*Failure after 12 months*	*Failure after 18 months*	*Failure after 24 months*
1st month	18	01 54P	00	00	00
2nd month	24	01 54P (Ga)	01 64P	00	00
3rd month	29	02 75O, 64P	00	01 74P	00
4th month	18	02 74P, 84P	01 55P	00	00
5th month	27	02 64P, 84P	00	00	00
6th month	12	01 54P	01 84P	00	00
Total	128	09	03	01	00

O: Occlusal surface pulpotomy; P: Proximal surface pulpotomy, GA: tooth treared under general anesthesia

**Table 3:** Areas of failure

*Areas of failure*	*No. of teeth*
Clinical abscess+ mobility + furcation radiolucency	02
Clinical abscess + mobility + periapical radiolucency	04
H/o severe pain with furcation and periapical radiolucency	06
Internl resorption	01
Total	13

Cotton roll isolation is used in all patients. As mentioned earlier we have not used rubber dam application to four patients who were willing to accept it to avoid creating a small group of variables. Although rubber dam is recommended to provide a saliva-free working area in endodontic treatment, but there are ample kinds of literature which concluded that careful cotton roll isolation does not have much effect in short and long term outcome of restorative procedure when compared to rubber dam application in child patient.^18,20–23^

Composites with glass ionomer base are chosen for occlusal restorations as better moisture control can be achieved in this area while using cotton roll isolation, especially in the lower arch. The self-etch adhesive was used to reduce working time and avoid washing in cotton roll isolation. RMGI used for proximal restorations is to avoid a two-step procedure of glass ionomer and composite as the chance of moisture contamination is more in this area while using cotton roll isolation, especially in the lower arch.

Only 13 cases of failure were observed in a period of 2 years of follow-up. It has been observed that most of the failures occurred in the first 6 months. Once the period after pulpotomy procedure crossed one year, there is no failure observed except one tooth. All the failures are attributed to infection of the radicular pulp which ultimately leads to apical or furcal radiolucency and lateral abscess in some cases. One tooth showed signs of internal resorption in the radiograph.

The main advantage of this procedure is that it saves the tooth structure, so no SSC placement is required. It has been observed that saving a larger amount of occlusal surface structure can provide a primary tooth enough strength against post-pulp therapy crown fracture so that application of SSC can be avoided.^24–26^ In our study, no crown fracture in the period of 2 years of observation corroborates with those findings. We have observed that this nonconventional formocresol pulpotomy procedure has three fold advantages over conventional formocresol pulpotomy. Firstly, saving tooth structure certainly excludes the necessity of providing an SSC. Second, cutting a lesser amount of tooth structure naturally saves some time and third, viewing the previous two benefits the procedure is certainly cost and time effective. We found it as an excellent approach to pulpotomy procedure that saves time and cost. It is also a very useful procedure while dealing uncooperative children and also a sensible approach where we undertake multiple pulpotomies in a single visit, especially treating patients under general anesthesia.

## CONCLUSION

In our study, it has been observed that formocresol pulpotomy done with quickly made smaller access to the pulp chamber is equally successful than that of conventional formocresol pulpotomy procedure. It is also observed that saving of the tooth structure by making smaller access can eliminate the necessity of post pulpotomy placement of an SSC. Working with lesser chair side time is an important aspect in the management of pediatric dental patients. If treatment like pulpotomy can be successfully done in a shorter time and lesser appointments as no appointments required for placement of SSC, a lot more children; who are unable to cooperate in dental clinic and at times not considered fit for this kind of treatment on OPD dental chair, can be brought under this very useful treatment procedure without using sedation or general anesthesia.
